# Reliability of Simple Reaction Time Measurement During Single-Leg Standing in Patients After Primary Anterior Cruciate Ligament Reconstruction

**DOI:** 10.7759/cureus.80051

**Published:** 2025-03-04

**Authors:** Shunsuke Ohji, Junya Aizawa, Kenji Hirohata, Takehiro Ohmi, Tomoko Kawasaki, Hideyuki Koga, Kazuyoshi Yagishita

**Affiliations:** 1 Clinical Center for Sports Medicine and Sports Dentistry, Institute of Science Tokyo, Tokyo, JPN; 2 Department of Physical Therapy, Juntendo University, Tokyo, JPN; 3 Sports Science Center, Institute of Science Tokyo, Tokyo, JPN; 4 Department of Joint Surgery and Sports Medicine, Graduate School of Medical and Dental Sciences, Institute of Science Tokyo, Tokyo, JPN

**Keywords:** accuracy, aclr, anterior cruciate ligament reconstruction, reaction time, reliability

## Abstract

Objective

Compared to healthy athletes, athletes who have undergone anterior cruciate ligament (ACL) reconstruction have been shown to have delayed reaction time (RT). However, the reliability of RT measurements after ACL reconstruction remains unknown, and no study has measured RT after ACL reconstruction in the single-leg standing position. This study aimed to validate the reliability of simple RT measurement in a single-leg standing position within a single session after ACL reconstruction.

Materials and methods

Participants who underwent ACL reconstruction were recruited for this study. A response-measuring instrument synchronized with a mat sensor measured simple RT in the single-leg standing position. The participant assumed the single-leg standing position on the mat sensor and lifted the sole off the mat sensor as soon as possible after the lamp lighting. The RT was defined as the time from the lamp lighting to when the sole left the mat sensor. The RT of each operative and nonoperative side was measured 10 times randomly. After confirming the normality of the data, the frequency effect on the 10 trials was confirmed by repeated measures analysis of variance.

Results

The mean of 10 RT trials in the single-leg standing position was 0.444 ± 0.072 s on the operative side and 0.436 ± 0.060 s on the nonoperative side. Repeated measures analysis of variance showed no significant difference between measurements for either the operative or nonoperative side. After the second trial, the intraclass correlation coefficient exceeded 0.900 for both the operative and nonoperative sides. The standard error of each measurement for the three trials was 0.006 s and 0.041 s for the operative and nonoperative sides, respectively.

Conclusion

The reliability of measurement within a single session of simple RT in the single-leg standing position after ACL reconstruction was acceptable.

## Introduction

Anterior cruciate ligament (ACL) injury is a common sports injury. Active patients who participate in jumping, cutting, and pivoting sports often undergo ACL reconstruction to avoid the risk of secondary menisci or cartilage damage [1]. However, even after ACL reconstruction and rehabilitation, functional disabilities such as limited joint range of motion, muscle weakness, and poor jump landing performance remain [2-4].

One of the functional impairments that occur after ACL reconstruction is a delayed reaction to stimuli. A reaction is the neurocognitive function to respond rapidly and appropriately to unexpected or controlled environmental stimuli. Reaction time (RT) is assessed in injured individuals, including those who are post-ACL reconstruction patients, to evaluate the speed of processing responses to stimuli and motor behavior [5]. However, it has been noted that existing tests do not predict a safe return to sport after ACL reconstruction [6-8]. This limitation stems from the fact that standard return-to-sport tests primarily assess physical performance and omit neurocognitive tasks, such as reacting to stimuli, which are crucial during sports activities [9]. To date, no studies have demonstrated an association between delayed RT and performance or re-injury after ACL reconstruction. This gap highlights the need to develop an RT test that evaluates response to stimuli post-ACL reconstruction.

Previous studies have shown that patients after ACL reconstruction exhibit delayed RTs for seated knee extension movements and standing forward steps to light stimulation compared to controls [10,11]. However, there are three key considerations when measuring RT in this context. First, the reliability of the RT test has yet to be validated. RT, typically captured within a few hundred milliseconds, requires thorough verification of both relative and absolute reliability. Secondly, the nature of RT measurement itself warrants consideration. In a study by Kaneko et al. [10], the task involved measuring RTs for knee extension movements against a light stimulus while seated, a task that may be overly simplistic for athletes engaged in sports [11]. Armitano-Lago et al. [11] described a task requiring participants to rapidly step forward with a single leg in response to a light stimulus while being in a double-leg standing position. This task involves weight shifting to the supporting leg, moving from a stable double-leg standing position to a single-leg standing position for the step [12]. This complexity is exemplified by findings from another study, which reported a delayed onset of muscle activity in low extremity muscles when transitioning from double-leg to single-leg standing position in the ACL reconstruction group compared to controls [13], complicating the interpretation of RT delays in these combined functions (step leg response + shifting weight of the supporting leg). Finally, the optimal number of measurements for these tests remains unclear. It is pertinent to confirm the appropriate number of measurements and consider potential learning effects associated with repeated testing.

Based on the above, we considered measuring the RT in the single-leg standing position desirable after ACL reconstruction. Measuring the RT of a single leg may enable the assessment of simple RT, as it eliminates compensatory movements on the nonoperative side. Thus, we aimed to validate the reliability of measuring simple RT in a single-leg standing position within a single session after ACL reconstruction. Additionally, we examined the number of trials needed to ensure reliability. We hypothesized that measuring simple RTs in the single-leg standing position would demonstrate acceptable reliability.

## Materials and methods

Research methodology

This cross-sectional study examined the reliability of the newly developed simple RT test in a single-leg standing position. Demographic and surgical data were obtained from the medical records. Demographic data, including age, sex, and activity level, were measured using a modified Tegner activity scale [14]. Surgical data included graft type, meniscal repair presence, and the months from measurement to surgery. Ethical approval was obtained from the review board of the Institute of Science Tokyo (approval number: M2021-191). This study was conducted using the principles of the Declaration of Helsinki, and written informed consent was obtained from all participants.

Participants

Participants who underwent primary ACL reconstruction were included if they met the following criteria: 1) age of 16-45 years at the time of measurement [15,16]; 2) ≥6 months post-surgery; 3) participation in sports with a modified Tegner activity scale score [14] ≥5 before ACL injury; 4) autografts sourced from the hamstrings or bone-patellar tendon-bone; and 5) no pain, knee instability, or other problems in single-leg static balance and RT measurement. All participants underwent postoperative rehabilitation based on a previous study [17]. Participants were excluded from recruitment based on the following criteria: 1) having undergone any surgery other than ACL reconstruction within 6 months before reconstruction; 2) having undergone multiple ligament reconstruction and lateral extra-articular tendon lengthening; 3) having cartilage damage requiring surgical intervention; 4) having had previous ACL reconstruction, or 5) <6 months post ACL reconstruction surgery.

Measurement of simple RT in a single-leg standing position

**Figure 1:**
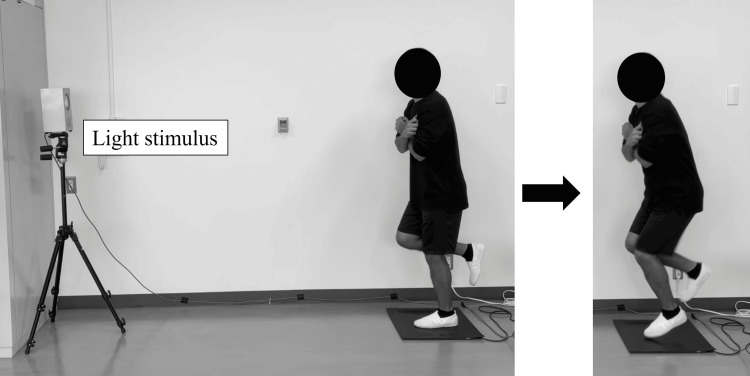
Measurement of single-leg simple reaction time The reaction time is measured as the time it takes for the participant's foot to leave the mat sensor from the start of the light stimulus.

Measurements of simple RT in a single-leg standing position were conducted in a quiet laboratory [18]. A modified version of the response-measuring instrument (T.K.K1264p, Takei Kiki Kogyo, Japan) was used for the measurements. A lamp lighting platform was placed 2 m from the front edge of the mat sensor. The lamp lighting position was set at a height of 150 cm from the ground to ensure visibility for all the participants (Figure 1). The measurement frequency was 1000 Hz. The lamp was set to light randomly within 10 s from the start of the measurement. The starting position was the single-leg standing position, with the non-support leg flexed at 45° at the hip joint and 90° at the knee joint. The participants were allowed a slight flexion of the lower limb of the supporting leg. Both arms were crossed in front of the chest to control the upper extremity influence. The participant assumed the starting position on the mat sensor and moved the lower leg away from the mat sensor as quickly as possible after the lamp lighting. The time from lamp lighting to the time the measuring leg was completely off the mat sensor was defined as the RT. The order of measurement for the operative and nonoperative sides was randomized. These were randomized using a Microsoft Excel randomized function (Microsoft Corporation, Redmond, USA). Ten trials were conducted after two or three practice sessions, each before measurement.

Statistical analysis

The normality of the RT data was confirmed using the Shapiro-Wilk test [19]. The difference between the 10 trials was confirmed by repeated measures analysis of variance (ANOVA) [20]. To confirm relative reliability, the intraclass correlation coefficient (ICC) was calculated from two to 10 trials. The ICC levels were set to almost perfect (0.81-1.00), substantial (0.61-0.80), moderate (0.41-0.60), fair (0.21-0.40), and slight (0.0-0.20) [21]. The standard error of the mean (SEM) was calculated to confirm absolute reliability. Although increasing the number of trials may improve measurement accuracy, conducting 10 trials is not realistic in a clinical setting. Therefore, if the ICC of three trials, which is the minimum number needed to maintain reliable measurements while ensuring clinical feasibility, was almost perfect, the representative value of three trials for each participant was calculated. Statistical significance was set at p<0.05. Statistical analyses were performed using SPSS ver. 28 (IBM Corp., Armonk, USA).

## Results

Ten patients participated in this study (male, n=5; age, mean±standard deviation 23.6±5.1 years; months from surgery to measurement, 12.1±5.8) (Table 1). None of the included participants had any previous lower limb injuries that could potentially influence reaction time measurements, other than the ACL injury requiring reconstruction.

**Table 1:** Participants’ descriptive data

Demographic variables	
Sex (female/male), n	5/5
Age, year	23.6±5.1
Height, cm	166.7±12.3
Body weight, kg	68.9±16.4
Body Mass Index, kg/m^2^	24.5±3.3
Months from surgery to measurement	12.1±5.8
Preoperative modified Tegner activity score	7.8±1.4
Graft type (hamstring/bone-patellar-tendon-bone), n	9/1
Meniscus repair (Yes/No), n	7/3

Representative values and transitions of trials for each participant's 10 trials are shown in Table 2. The mean of 10 trials of single-leg standing position RT was 0.444±0.072 s on the operative side and 0.436±0.060 s on the nonoperative side.

**Table 2:** Representative values from 10 trials for each participant SD, standard deviation; SEM, standard error of measurement

	Operative side			Nonoperative side		
Participants	Mean value	SD	SEM	Mean value	SD	SEM
A	0.426	0.023	0.007	0.416	0.032	0.010
B	0.394	0.018	0.006	0.374	0.016	0.005
C	0.432	0.049	0.015	0.441	0.032	0.010
D	0.497	0.027	0.009	0.526	0.059	0.019
E	0.623	0.040	0.013	0.558	0.033	0.010
F	0.445	0.026	0.008	0.419	0.029	0.009
G	0.410	0.025	0.008	0.404	0.019	0.006
H	0.430	0.022	0.007	0.432	0.023	0.007
I	0.360	0.018	0.006	0.381	0.031	0.010
J	0.418	0.023	0.007	0.410	0.027	0.009
All participants	0.444	0.072	0.023	0.436	0.060	0.019

Repeated measures ANOVA showed no significant difference between 1-10 trials for either the operative or nonoperative side (Table 3).

**Table 3:** Descriptive statistics and results of repeated measures ANOVA for reaction time (sec) for each trial T, Trial; SD, standard deviation; IQR, Interquartile range

Operative side											
	T1	T2	T3	T4	T5	T6	T7	T8	T9	T10	
Mean±SD	0.456±0.08	0.446±0.09	0.436±0.08	0.451±0.07	0.443±0.08	0.446±0.10	0.448±0.08	0.045±0.08	0.430±0.06	0.432±0.06	P > 0.05
Median (IQR)	0.440 (0.10)	0.425 (0.10)	0.419 (0.10)	0.440 (0.07)	0.413 (0.08)	0.436 (0.09)	0.443 (0.06)	0.440 (0.09)	0.415 (0.05)	0.413 (0.04)	
Nonoperative side											
	T1	T2	T3	T4	T5	T6	T7	T8	T9	T10	
Mean±SD	0.452±0.07	0.457±0.09	0.458±0.08	0.428±0.07	0.426±0.07	0.421±0.05	0.415±0.06	0.426±0.05	0.438±0.067	0.440±0.05	P > 0.05
Median (IQR)	0.435 (0.06)	0.419 (0.14)	0.438 (0.10)	0.412 (0.11)	0.410 (0.07)	0.404 (0.06)	0.390 (0.10)	0.430 (0.05)	0.413 (0.07)	0.429 (0.08)	

The ICC was almost perfect after the second trial, exceeding 0.900 on both the operative and nonoperative sides (Table 4).

**Table 4:** Intraclass correlation coefficients for the 2 to 10 trials ICC, intraclass correlation coefficients; CI, confidence interval; SEM, standard error of measurement of each trial. * P < 0.001

	Operative side			Nonoperative side	
	ICC (95%CI)	SEM		ICC (95%CI)	SEM
ICC (1, 2)	0.917 (0.686-0.979)*	0.030		0.932 (0.742-0.983)*	0.019
ICC (1, 3)	0.959 (0.882-0.989)*	0.019		0.909 (0.743-0.975)*	0.022
ICC (1, 4)	0.964 (0.907-0.964)*	0.017		0.924 (0.805-0.979)*	0.019
ICC (1, 5)	0.969 (0.924-0.991)*	0.015		0.945 (0.866-0.984)*	0.016
ICC (1, 6)	0.978 (0.947-0.994)*	0.013		0.950 (0.881-0.986)*	0.014
ICC (1, 7)	0.982 (0.959-0.995)*	0.012		0.959 (0.904-0.988)*	0.013
ICC (1, 8)	0.986 (0.967-0.996)*	0.010		0.962 (0.912-0.989)*	0.012
ICC (1, 9)	0.984 (0.963-0.995)*	0.011		0.970 (0.932-0.991)*	0.011
ICC (1, 10)	0.984 (0.964-0.995)*	0.010		0.971 (0.935-0.992)*	0.010

The individual variability of the three-trials is shown in Table 5. For both the operative and nonoperative sides, the SEM (min-max) ranged from 0.006 to 0.041 s.

**Table 5:** Representative reaction time values (sec) from three trials for each participant SD, standard deviation; SEM, standard error of measurement

	Operative side			Nonoperative side		
Participants	Mean value	SD	SEM	Mean value	SD	SEM
A	0.429	0.014	0.009	0.405	0.044	0.025
B	0.400	0.050	0.014	0.393	0.010	0.006
C	0.439	0.128	0.041	0.459	0.050	0.029
D	0.499	0.037	0.011	0.586	0.070	0.041
E	0.635	0.034	0.010	0.588	0.040	0.023
F	0.476	0.021	0.006	0.448	0.032	0.019
G	0.394	0.040	0.012	0.410	0.020	0.012
H	0.443	0.019	0.006	0.449	0.024	0.014
I	0.345	0.026	0.009	0.409	0.033	0.019
J	0.396	0.045	0.013	0.410	0.006	0.003

## Discussion

Summary of the results

This study aimed to confirm the reliability of a single session of simple RT for a single leg after ACL reconstruction. There have been no previous reports on the measurement reliability of RT after ACL reconstruction or RT for tasks performed using only a single leg. This study demonstrated sufficient reliability for the measurement of RT after ACL reconstruction.

Reliability of measurement, learning effects, and individual variability

The results from the repeated measures ANOVA indicated no statistical differences between the measurement counts. This result suggests a minimal learning effect on the single-leg standing simple RT measurements for the participants in this study. It would be acceptable to commence formal measurements after two or three practice sessions.

In this study, the ICCs after two trials within a single session were almost perfect (>0.900) for both the operative and nonoperative sides. However, the lower limit of the 95% confidence interval for the ICC of the two trials was low (0.686 for the operative side and 0.742 for the nonoperative side). Ten trials are not realistic in clinical practice, and it is desirable to perform as few trials as possible, with the highest possible reliability. Considering the ICC reference value [21] and the results of this study, a more reliable evaluation could be performed by using the average of three or four trials. The SEM for individuals with three trials is 0.006-0.041 s for both the operative and nonoperative sides (table 5), and this range may be viewed as individual variability.

Limitations

Since this study analyzed participants who responded to recruitment within the study institution, it did not include all patients from the study period, raising the possibility of selection bias.

Additionally, our study has limitations regarding the sample characteristics. The relatively small sample size may limit the generalizability of our findings, and a larger cohort would be beneficial for future validation studies. The participants in this study also represented a relatively narrow height spectrum (mean±SD: 166.7±12.3 cm), which might not fully represent the variety of patient populations encountered in clinical settings. While this homogeneity in height might have contributed to the consistency of our measurements, it also suggests that the reliability of RT measurements for patients outside this height range needs to be investigated separately.

Furthermore, we focused solely on measuring reliability within a single session. Thus, the reliability of retests and minimal detectable changes when measurements are taken at different times are not known. Study participants were assessed at a mean of 12.1 months postoperatively, with a wide range from 6 to 25 months. This variation in postoperative duration may have influenced participants' reaction times, as functional improvement could differ depending on the time elapsed since surgery. Therefore, it is necessary to clarify the participants and timing of the measurements when applying the results of this study.

Although the participants in this study confirmed that they had no problems with single-leg static standing and single-leg RT measurements, they did not perform detailed balance assessments such as the center of pressure (COP). Previous studies have shown that COP velocity during single-leg static standing and single-leg squatting is higher after ACL reconstruction compared to the control group [22,23]. The possibility of an error in the RT measurement results cannot be ruled out in cases of extreme impairment of single-leg static standing balance due to ACL injury and/or reconstruction.

Future perspective

Given that the current tests are insufficient for predicting a safe return to sports after ACL reconstruction, it is pertinent to develop a reliable assessment of response to stimuli after ACL reconstruction, such as the RT test. Future applications of this test should aim to validate the timing and muscle activity characteristics of RT against those of healthy adults. Additionally, factors including age, sex, graft type, and cognitive and physical functions, which may influence RT, need to be considered in future studies. Finally, we plan to measure simple RT in the single-leg standing position after ACL reconstruction to evaluate its correlation with the likelihood of returning to sports and the risk of re-injury.

## Conclusions

The reliability of measurement within a single session of simple RT in the single-leg standing position after ACL reconstruction was acceptable. Future studies will accumulate a reasonable sample size to characterize the asymmetry of RT in the single-leg standing position after ACL reconstruction.
